# Paediatric tuberculosis diagnosis using *Mycobacterium tuberculosis* real-time polymerase chain reaction assay: a systematic review and meta-analysis

**DOI:** 10.1186/s13643-021-01836-w

**Published:** 2021-10-27

**Authors:** Emmanuel Oladipo Babafemi, Benny P. Cherian, Beatrice Ouma, Gilbert Mangua Mogoko

**Affiliations:** Liverpool John Moores University-City Campus, Liverpool, Merseyside UK

**Keywords:** Paediatric Tuberculosis, *Mycobacterium tuberculosis*, Real-time polymerase chain reaction-based assay, Pulmonary samples, Extra-pulmonary samples, Systematic review, Meta-analysis

## Abstract

**Background:**

Rapid and accurate diagnosis of paediatric tuberculosis (TB) is key to manage the disease and to control and prevent its transmission. Collection of quality sputum samples without invasion methods from paediatrics (age < 16 years) with presumptive pulmonary tuberculosis (PTB) remains a challenge. Thus, the aim of this meta-analysis was to assess the overall accuracy of a real-time polymerase chain reaction (RT-PCR)-based assay, for routine diagnosis of MTB in different samples from paediatrics with active pulmonary and extra-pulmonary tuberculosis using mycobacterial culture as the gold standard in clinical microbiology laboratories.

**Methods:**

We conducted a systematic review and meta-analysis to examine the diagnostic test accuracy of RT-PCR based assay for the detection of MTB in paediatric clinical samples.

A systematic literature search was performed for publications in any language. MEDLINE via PubMed, EMBASE, and Web of Science were among 9 bibliographic databases searched from August 2019 until November 2020. Bivariate random-effects model of meta-analysis were performed to generate pooled summary estimates (95% CIs) for overall accuracy of RT-PCR based assay compared to mycobacterial culture as the reference standard.

**Results:**

Of the 1592 candidate studies, twenty-one eligible studies met our inclusion criteria. In total, the review and meta-analysis included 5536 (3209 PTB and 2327 EPTB). Summary estimates for pulmonary TB (11 studies) were as follows: sensitivity 56 (95% CI 51–62), specificity 97 (95% CI 96–98) and summary estimates for extra-pulmonary TB (10 studies) were as follows: sensitivity 87 (95% CI 82-91)) specificity 100 (95% CI 99–100). There was significant heterogeneity in sensitivity and specificity among the enrolled studies (*p* < 0.001).

**Conclusions:**

Our results suggested that the RT-PCR based assay could be a useful test for the diagnosis of paediatrics TB with high sensitivity and specificity in low-income/high-burden and upper medium income/low-burden settings. From the study, RT-PCR assay demonstrated a high degree of sensitivity for extra-pulmonary TB and good sensitivity for pulmonary TB which is an important factor in achieving effective global control and for patient management in terms of initiating early and appropriate anti-tubercular therapy.

**Systematic review registration:**

PROSPERO CRD42018104052

**Supplementary Information:**

The online version contains supplementary material available at 10.1186/s13643-021-01836-w.

## Background

Paediatric tuberculosis (PaeTB) diagnosis presents a major challenge [1]. Tuberculosis (TB), an infectious disease caused by the bacillus *Mycobacterium tuberculosis* (MTB), is spread from person to person predominantly through an airborne route and remains a major global health problem as it causes ill-health among millions of people [2]. TB is the leading cause of death from a single infectious agent (ranking above HIV/AIDS) and about a quarter of the world’s population is infected with MTB [3]. According to the World Health Organization, globally, an estimated 10.0 million (range, 8.9–11.0 million) people fell ill with TB in 2019, a number that has been declining very slowly in recent years. However, World Health Organization (WHO) estimates that annually, 1.2 million children have TB disease and many more harbour a latent form of infection [3]. The disease typically affects the lungs (pulmonary TB) but can also affect other sites (extra-pulmonary TB).

This review is important because diagnosis of PaeTB disease in children can be challenging as MTB can only be detected in biologic samples from fewer than 50% of children with TB [4, 5]. MTB isolation is difficult due to the paucibacillary nature of the disease hence diagnosis often relies on clinical, epidemiological, radiological, and tuberculin skin test. TB in children is often missed or overlooked due to non-specific symptoms and or non-specific diagnostic tests [1, 6]. Ninety-four percent of children with TB are treated empirically in TB high-burden countries with non-specific diagnostic tests [7].

The main challenges and issues which this review aimed to address are lack of accurate estimates due to under-recognition, challenges in diagnosis and non-existent of an accurate diagnostic test to confirm TB in children [8]. The lack of a simple and effective diagnostic test that can be utilised in resource-limited settings, where the infection is endemic, has hindered its control [9]. It is rarely bacteriologically confirmed [10]. Young children are at particular risk of developing severe, often fatal, or life-long disabling forms of TB. In 2019, approximately 230,000 children died of TB, among whom 52,000 were living with HIV (http://www.who.int/tb/publications/global_report/en). PaeTB remains a major cause of morbidity and mortality globally, particularly in developing countries. Most deaths from PaeTB could be prevented with early diagnosis and appropriate treatment [11]. The actual burden of TB in children is likely higher given the challenge in diagnosing childhood TB in many low-income countries where the diagnosis of paediatric TB is solely based on clinical evidence and smear microscopy [10]. These are more difficult in paediatric population as they are unable to produce deep cough for adequate sputum [12]. Gastric aspirate as a sample has the drawbacks that it is minimally invasive and requires fasting state [13]. However, stool as a sample for intrathoracic tuberculosis has been explored on the premise that children usually swallow their sputum and it is convenient to obtain, non-invasive compared to sputum or gastric aspirate [14].

Latent tuberculosis infection (LTBI) is defined as a state of persistent immune response to stimulation by MTB antigens with no evidence of clinically manifest active TB [15]. It is estimated that the lifetime risk of an individual with LTBI for progression to active TB disease is 5–10% over their lifetime [16]. This risk is particularly high among children under the age of 5 years [17]. Tuberculin skin test (TST) or interferon-gamma release assay (IGRA) can be used to test for LTBI, as there is no ‘gold standard’ test for LTBI [18]. It is only a marker of exposure rather than disease [1]. Establishing an accurate diagnosis of PaeTB in children can be more difficult than adult TB, because of the challenge children have in expectorating good-quality sputum or absence of lung parenchymal disease as in primary complex [19]. In children, culture methods have a greater, yet highly variable, sensitivity which improve diagnosis but takes between 2 and 8 weeks in most cases and the sputum sample lacks representative of lower respiratory tract [20, 21]. Other diagnostic approaches are based on clinical presentations, imperfect tools such as radiology which is subject to inter-observer variability to detect radiographic abnormalities, contact history, and tuberculin skin test, all of which are of low specificity [22].

Given the difficulty in diagnosing TB disease in paediatrics this systematic review assesses all the available published primary research studies to provide summary estimates to contribute to rapid and accurate diagnosis of PaeTB using RT-PCR based assays which can improve diagnostic accuracy for diagnosing MTB infection in paediatrics with tuberculosis compared to the mycobacterial culture-based assays. RT-PCR assays are nucleic acid amplification tests (NAATs), which was developed in 1983, and are now a common tool for the rapid diagnosis of many infectious diseases, including PaeTB [23–26]. The assay has much better accuracy than sputum smear microscopy [27]. Due to advances in technologies over the past decades, the TB diagnostics pipeline has improved tremendously showing promise [28, 29]. RT-PCR assay is commonly used to determine whether DNA or a unique sequence of the MTB is present in a sample and detects amplified DNA as the reaction progresses in real time [30]. Xpert MTB/RIF (Xpert) (Cepheid, USA), an automated cartridge-based RT-PCR assay, is currently recommended by the World Health Organization (WHO) as the initial diagnostic test in presumptive PTB cases for adults and children [31].

The outputs of this systematic review will serve as a resource for decision-makers, providing government stakeholders and healthcare practitioners with the tools to make evidence-based decisions for PaeTB diagnosis and control. It summarises current evidence-based clinical practice that can help to develop future guidelines and healthcare policy when choosing the most appropriate tool for rapid and accurate detection of MTB by RT-PCR assay in paediatric clinical samples on routine basis.

## Methods

This review was registered and is in accord with the standardised written protocol (systematic review registration with the International Prospective Register of Systematic Reviews (PROSPERO) database PROSPERO CRD42018104052) that followed the PRISMA-P (Preferred Reporting Items for Systematic Reviews and Meta-Analyses Protocols) statement guidelines [32]. Additional file 1 shows the PRISMA checklist. The published protocol can be accessed on https://systematicreviewsjournal.biomedcentral.com/articles/10.1186/s13643-019-1137-y. Quality of included studies was assessed by Quality Assessment of Diagnostic Accuracy Studies-2 (QUADAS-2) [33]. Institutional ethical review approval was not needed for this review.

### Strategy

#### Electronic searches

Search terms (“tuberculosis”, mycobacterium tuberculosis, extrapulmonary tuberculosis, pulmonary tuberculosis, paediatric tuberculosis), “Real-time polymerase chain reaction”, real-time pcr, real-time pcr assay, “rt-pcr”, “Nucleic Acid Amplification Test”, “NAAT”, “culture-based media”, culture-based assay, “liquid media”, “solid media”, “paediatric”, “paediatrics”, “children”) were used to generate a list of primary studies in any language with no restriction on date of publication, and publication status (see Additional file 2 for search terms). There was no restriction regarding the language, date of publication and publication status. Studies that recruited children less than 16 years of age being investigated for MTB infection using RT-PCR assay across lower- and middle-income countries (LMICs), and Upper middle-income countries (UMICs) accompanied by mycobacteriological culture investigation as the reference standard were included to achieve a more reliable estimate of diagnostic accuracy which is important to ensure that the process of identifying studies is as thorough and unbiased as possible.

Two investigators (EB, BC) independently and systematically carried out the search. Searches using electronic bibliographic databases (MEDLINE via PubMed, EMBASE, LILACS, BIOSIS Citation Index, Web of Science, SCOPUS, ISI Web of Knowledge, Cochrane Infectious Diseases Group Specialised Register (CIDG SR), Cochrane Registry of Diagnostic Studies, National Institute for Health Research, PROSPERO, Google Scholar Turning Research into Practice (TRIP) took place in August 2019 and was updated in November 2020. The MEDLINE search strategy is outlined in Additional file 2. The MEDLINE search was imported to EMBASE, Cochrane Infectious Diseases Group Specialised Register and other databases to identify additional records [34, 35]. The search strategy for each database was validated by a librarian information specialist familiar with the topic.

Attempts were made to avoid missing relevant studies by searching other sources such as reference lists of relevant reviews, selected studies, portal of the WHO International Clinical Trials Registry Platform (www.who.int/trialsearch) to identify ongoing trials, as well as StopTB Partnership’s New Diagnostics Working Group (www.stoptb.org/wg/new_diagnostics/), the World Health Organization and Centers for Disease Control and Prevention websites, and proceedings of the International Union Against Tuberculosis and Lung disease (UNION) conference. A search of grey literature including conference proceedings (Conference Proceedings Citation Index–Science (CPCI-S)), Dissertations and Theses (www.proquest.com), and expert information was sought and added to our resource material.

Besides full articles, abstracts, and letters to the editor with sample sizes > 20 was also considered for inclusion. There was no language limitation to the search. Abstracts or articles in languages other than English were screened using ‘Google Translator’.

### Inclusion and exclusion criteria

Study designs such as observational, cross-sectional studies, cohort studies (prospective and retrospective) and case-control designs for the detection of MTB from paediatrics clinical samples of age < 16 years were eligible for inclusion if the studies (1) compared RT-PCR based assay to a reference/gold standard method— MTB culture-based (either liquid or solid) assay, (2) described original research, (3) reported total number of patients tested and positive/negative results that allowed calculation of true positives (TP), true negatives (TN), false positives (FP) and false negatives (FN). Studies were excluded if (1) RT-PCR assay was not used in the study, (2) if age of participants is > 16 years, (3) all samples were not tested by reference/gold standard test—MTB culture-based (either liquid or solid) assay, (4) reference test was a combination of greater than one diagnostic test, (5) it included animal studies, (6) RT-PCR based assay was used for detecting non-tuberculosis mycobacteria, (7) RT-PCR based assay was used for detecting MTB from clinical isolates and not the pathological specimens/samples and (8) possible duplicate publication, when an author published more than one study. The existence of overlapping study populations was ascertained by checking sample recruitment sites and/or periods. The article reporting on the largest number of samples was included in our study.

#### Selection of studies

Full-text articles were screened independently (by EB and BC), using a PRISMA flow chart [32] for eligibility for use in the study to minimise bias in selection. Any disagreements were resolved through discussion and where needed, by a third reviewer (BO). Any rejected studies were documented.

#### Data extraction

Data extraction were independently carried out by EB and BC from each selected study using a predetermined list of categories/characteristics: participants/population, country, index test, reference test, disease and target sequence for detection of MTB DNA in PaeTB (Table 1).

**Table 1:** Characteristics of the included studies

Author year (***n***)	Country	Study design	Total number of samples (***N***)		Reference test: culture	Index test: RT-PCR	Target sequence
			PTB	EPTB			
Bates et al. (2013) [36]	Zambia-(L)	Prospective-descriptive	142		Liquid culture (MGIT)	RT-PCR Xpert MTB/RIF	*rpo*B probe
Bates et al. (2013) [36]	Zambia-(L)	Prospective-descriptive		788	Liquid culture (MGIT)	RT-PCR Xpert MTB/RIF	*rpo*B probe
Chipinduro et al. (2017) [37]	Zimbabwe-(L)	A cross-sectional		222 (stool)	LJ	RT-PCR Xpert MTB/RIF	*rpo*B probe
El Khechine et al. (2009) [38]	France-(U)	Diagnostic case-control	–	134	BACTEC 9000 MB LJ	RT-PCR(MX3000)	IS6110 gene
Gous et al. (2015) [39]	South Africa-(U)	Prospective	345	–	Liquid culture (MGIT)	RT-PCR Xpert MTB/RIF	*rpo*B probe
LaCourse et al. (2014) [40]	Malawi-(L)	Cross-sectional study	300	–	Bactec MGIT, BD)	RT-PCR Xpert MTB/RIF	*rpo*B probe
Memon et al. (2018) [41]	India-(L)	Diagnostic accuracy study	–	100	Bactec MGIT 960	RT-PCR Xpert MTB/RIF	*rpo*B probe
Mesman et al. (2019) [42]	Peru-(U)	Cohort study		259(stool)	BACTEC 9000 MB LJ	TruTip Mtb DNA	IS6110 real-time PCR
Nhu et al. (2013) [43]	Vietnam-(L)	Prospective		96	MGIT, Becton Dickinson)	RT-PCR Xpert MTB/RIF	*rpo*B probe
Nicol et al. (2011) [44]	South Africa-(U)	Prospective-descriptive	452	–	Liquid culture	RT-PCR Xpert MTB/RIF	*rpo*B probe
Nicol et al. (2013) [45]	South Africa-(U)	Prospective	–	115	Bactec MGIT 960	RT-PCR Xpert MTB/RIF	*rpo*B probe
Nicol et al. (2018) [46]	South Africa-(U)	Cohort study	367	–	MGIT, Becton Dickinson)	RT-PCR Xpert MTB/RIF	*rpo*B probe
Oberhelman et al. (2010) [47]	Peru-(U)	Prospective case-control study		218 (stool, GA, etc.)	LJ culture	hemi-nested IS6110 PCR	IS6110 PCR
Qing-Qin Yin et al. (2014) [48]	China-(U)	Prospective	255		Solid (LJ) and Liquid culture (Bactec MGIT 960	RT-PCR Xpert MTB/RIF	*rpo*B probe
Rachow et al. (2012) [49]	Tanzania-(L)	Prospective cohort study	164	–	Solid (LJ) and Liquid culture (Bactec MGIT 960	RT-PCR Xpert MTB/RIF	*rpo*B probe
Sekadde et al. (2013) [50]	Uganda-(L)	Cross-sectional diagnostic study	235	–	Solid (LJ) and Liquid culture (Bactec MGIT 960	RT-PCR Xpert MTB/RIF	*rpo*B probe
Walters et al. (2017) [51]	South Africa-(U)	Prospective		379 (stool)	BACTEC 9000 MB	RT-PCR Xpert MTB/RIF	*rpo*B probe
Wang et al. (2013) [52]	China-(U)	Retrospective	30	–	Bact/Alert 3D	LightCycler® 480 (Roche)	
Wolf et al. (2008) [53]	Peru-(U)	Diagnostic accuracy study	–	16 (6+) (stool)		hemi-nested IS6110 PCR	IS6110 PCR
Zar et al. (2012) [54]	South Africa-(U)	Prospective	535	–	Liquid culture (MGIT)	RT-PCR Xpert MTB/RIF	*rpo*B probe
Zar et al. (2013) [55]	South Africa-(U)	Prospective study	384	–	Bactec MGIT 960	RT-PCR Xpert MTB/RIF	*rpo*B probe

Key: *LJ* Löwenstein-Jensen, *Middlebrook 7H9 broth* liquid growth medium, *Middlebrook 7H11* Solid medium, *MGIT* mycobacterium growth indicator tube, *PTB* pulmonary TB, *EPTB* extra-pulmonary TB, *n* reference list number, *L* lower- and middle-income countries, *U* upper middle-income countries

### Assessment of study quality

The methodological quality for the included studies was assessed independently (EB and BC) according to the four domains (patient selection, index test, reference standard, and flow and timing) of the QUADAS-2 tool [33]. The study QUADAS-2 quality criteria are given in Additional file 3.

### Data synthesis and meta-analysis

We computed measures of test accuracy for each of the included studies using standard methods recommended for meta-analysis of diagnostic studies: sensitivity, specificity, positive likelihood ratio (PLR), negative likelihood ratio (NLR), diagnostic odds ratio (DOR) and 95% confidence intervals (CI) [56]. The 2 × 2 data (TP, FP, TN and FN) were extracted directly from the included studies. Where this information was not available, values were calculated from the data provided in the article. We used a DOR using the DerSimonian-Laird random-effect model to calculate and assess the overall accuracy. This model accounts for both within-study variability (random error) and between-study variability (heterogeneity) along with the area under the summary receiver operating characteristic (SROC) curve using the bivariate model [57, 58]. The bivariate model considers potential threshold effects and the correlation between binary tests (sensitivity and specificity). These measures were pooled using the random-effects model [57, 58]. Each of the included studies used in the meta-analysis contributed a pair of numbers: sensitivity and specificity. Since these measures are correlated, we summarised their joint distribution using a SROC curve. The SROC curve presents a global summary of test performance and shows the trade-off between sensitivity and specificity. A symmetric curve suggests that the variability in accuracy between studies is explained, in part, by differences in thresholds used by the studies. The area under the SROC curve is a global measure of overall performance of the test. An area under the curve value of 1 indicates perfect discriminatory ability of the test, while an area under the curve value of 0.5 means that the test does not have discriminating ability [57, 58].

Data were analysed using Meta-DiSC (version 1.4), Reviewing Manager ver. 5.4 (Cochrane Collaboration, Oxford, UK) [58, 59]. The data were displayed graphically on forest plots and SROC plots. The SROC curve was fitted using the Littenberg-Moses method [60].

We did not evaluate the publication bias because this is not usually recommended in the meta-analysis for diagnostic test accuracy [61]. Generally, a diagnostic accuracy study does not test a hypothesis; therefore, there is no *p* value for authors and publishers that may influence decisions about publication which are based on the statistical significance of the results [61, 62].

### Investigations of heterogeneity

We investigated heterogeneity because of its critical importance (1) to understand the possible factors that influence accuracy estimates and (2) to evaluate the appropriateness of statistical pooling of accuracy estimates using random-effects meta-analysis to generate sensitivity and specificity with 95% CIs from various studies [62].

The heterogeneities among the included studies were assessed visually using forest plots and SROC curves with 95% prediction regions and statistically with chi-squared (*χ*2) and using *I*-squared (*I*^2^) statistics with the following interpretation: *I*^2^ = 0, no heterogeneity; 0 < *I*^2^ < 25, mild heterogeneity; 25 ≤ *I*^2^ < 50, moderate heterogeneity; 50 ≤ *I*^2^ < 75, strong heterogeneity; 75 ≤ *I*^2^ < 90, considerable heterogeneity and 90 ≤ *I*^2^, extreme heterogeneity [61, 63].

Source of heterogeneity was investigated using stratified (subgroup) analyses. The following factors were specified a priori as potential sources of heterogeneity: impact of RT-PCR based assays on lower- and middle -income countries (LMICs) versus Upper middle-income countries (UMICs).

## Result

### Study characteristics

**Fig. 1:**
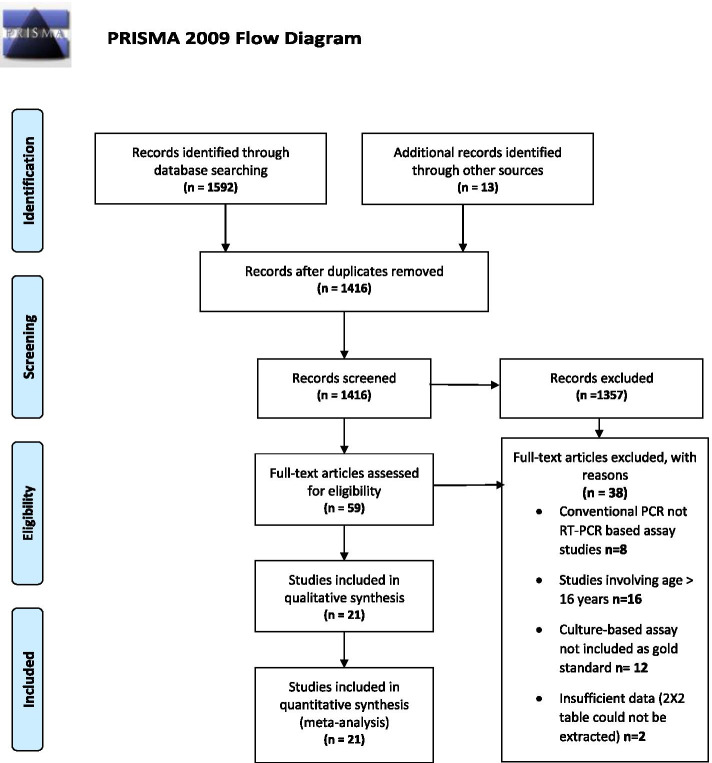
The Preferred Reporting Items for Systematic Reviews and Meta-Analyses (PRISMA)

Our search identified 1592 potentially relevant citations, of which 58 studies were selected based on relevance to the study topic. An additional 13 studies were identified from grey literature and references of full-text articles. After screening all the titles and abstracts, removing the duplicates and excluding the ineligible studies, 20 articles (5536 samples/patients) [36–55] were selected for full-text review and meta-analysis (Fig. 1).

Eleven [36, 39, 40, 44, 46, 48–50, 52, 54, 55] reported detection of pulmonary TB (PTB), 10 [36–38, 41–43, 45, 47, 51, 53] reported detection of extra-pulmonary TB (EPTB) and 1 [36] reported on both types of clinical sample. Table 1 summarises the main characteristics of the included studies. In total, the review and meta-analysis included 5536 (3209 PTB and 2327 EPTB) clinical samples obtained from 12 countries with high, moderate and low prevalence of PaedTB. There were 12 studies from the developed and eight studies from the developing countries. The studies included in the analysis were conducted in 11 different countries. Most studies (8 out of 20, 40%) were carried out prospectively [36, 39, 43–45, 48, 49, 51].

**Fig. 2:**
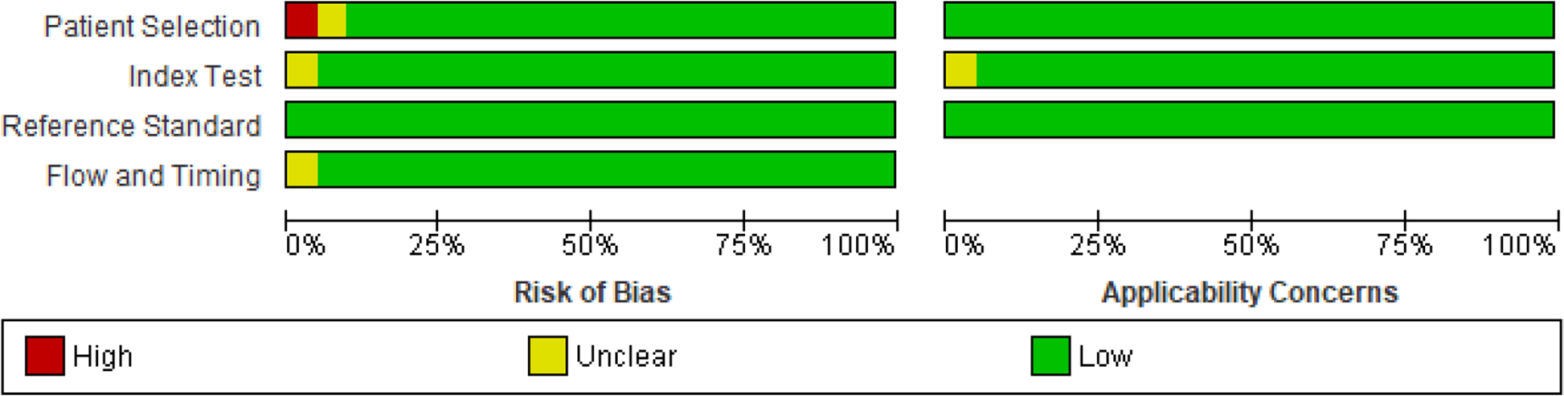
Risk of bias and applicability concerns graph: review authors’ judgements about each domain presented as percentages across included studies

**Fig. 3:**
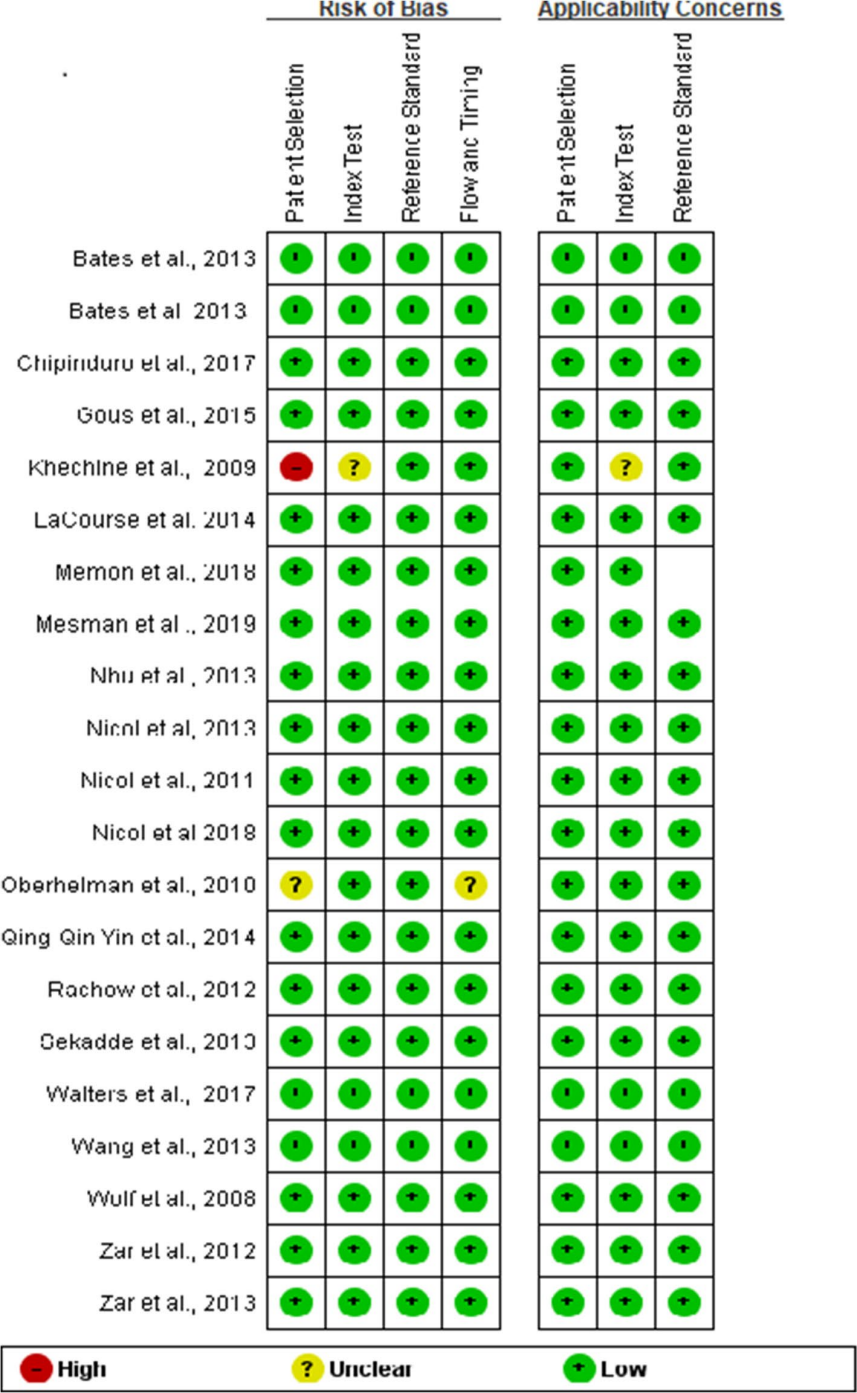
Risk of bias and applicability concerns summary: review authors’ judgements about each domain for each included study

Studies included paediatric patients with infections identified in primary, secondary and tertiary healthcare settings (see Table 1). Sixteen studies performed RT-PCR based assay using Xpert MTB/RIF on both paediatrics pulmonary and extra-pulmonary clinical samples [36, 37, 39–41, 43–46, 48–52, 54, 55] while 4 studies used other types of RT-PCR based assays to detect MTB from paediatrics extra-pulmonary (stool and gastric aspirates samples [36, 41, 47, 60]. Details of the RT-PCR based assays used are summarised in Table 1. The overall study quality assessed by the QUADAS-2 tool showed a low risk of bias except for studies using a case-control design (see Figs. 2 and 3).

The methodological quality of studies (assessed by the QUADAS-2 tool) was generally high, with 37 of the studies meeting all four domains of the criteria (see Figs. 2 and 3). All studies used assays that are based on RT-PCR principle as index test and culture-based assay as the reference test.

### Meta-analysis

Results as 95% CI values were as follows: overall sensitivity 56 (95% CI 51–62) and 87 (95% CI 82–91); 97 (95% CI 96–98) and 100 (95% CI 99–100) the values and confidence intervals for specificity are for PTB and EPTB samples, respectively. AUC was 0.98 and 0.99 for PTB and EPTB samples, respectively.

**Fig. 4:**
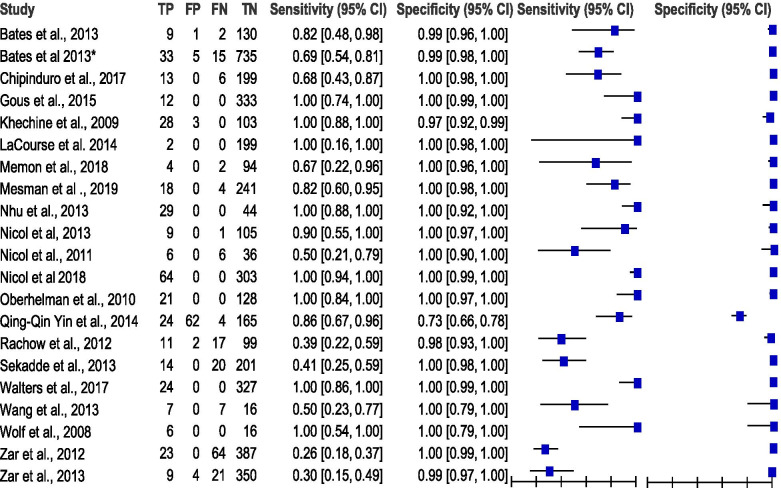
Forest plot of estimates of RT-PCR assay for pulmonary tuberculosis (PTB and EPTB). TP = true positive, FP = false positive, FN = false negative, TN = true negative. Between brackets are the 95% CI of sensitivity and specificity. The figure shows the estimated sensitivity and specificity of the study (blue squares) and its 95% CI (black horizontal line)

**Fig. 5:**
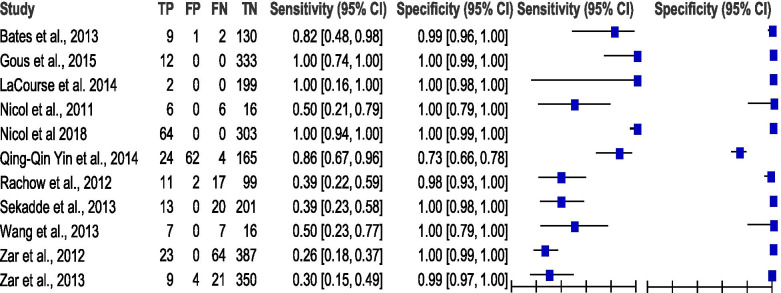
Forest plot of estimates of RT-PCR assay for pulmonary tuberculosis (PTB). TP = true positive, FP = false positive, FN = false negative, TN = true negative. Between brackets are the 95% CI of sensitivity and specificity. The figure shows the estimated sensitivity and specificity of the study (blue squares) and its 95% CI (black horizontal line)

**Fig. 6:**
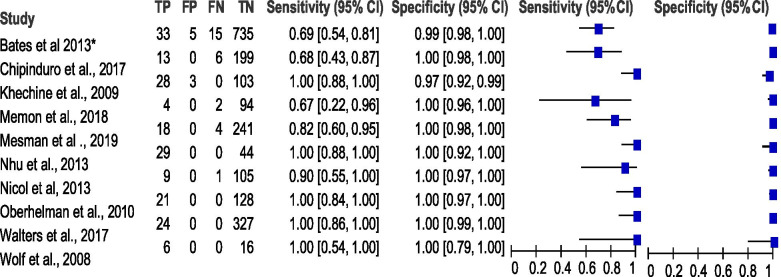
Forest plot of estimates of RT-PCR assay for extra-pulmonary tuberculosis (EPTB). TP = true positive, FP = false positive, FN = false negative, TN = true negative. Between brackets are the 95% CI of sensitivity and specificity. The figure shows the estimated sensitivity and specificity of the study (blue squares) and its 95% CI (black horizontal line)

The summary estimates of PTB for heterogeneity with chi-squared (*χ*2) using 95% CI for sensitivity, specificity, PLR, NLR and DOR were 151.22, 277.67, 205.09, 99.77 and 36.66 respectively, and *p* = 0 indicating significant heterogeneity across studies. *I*^2^ was between 72.7 and 93.4% showing significant heterogeneity. The summary estimates of EPTB heterogeneity with chi-squared (*χ*2) using 95% CI for sensitivity, specificity, PLR, NLR and DOR were 47.45, 19.19, 11.74, 29.44, and 13.02 respectively, and *p* < 0.5 indicating significant heterogeneity across studies. *I*^2^ was between 30.90 and 81.00% showing significant heterogeneity. There were considerable heterogeneities (see Table 2, Figs. 4, 5 and 6) in these data.

**Table 2:** Summary of statistical results for pulmonary tuberculosis (PTB) and extra-pulmonary tuberculosis (EPTB) clinical samples

Test property	Summary of measure test accuracy^a^ (95%)	Test of heterogeneity		
**PTB** **(*n*** **= 11;** ^b^**3209)** **AUC = 0.98**		*χ*2 (d.f. = 10)	*l*^2^	*p* value
Sensitivity	56(51–62)	151.22	93.4	< 0.001
Specificity	97(96–98)	277.67	96.4	< 0.001
Positive likelihood ratio (PLR)	70.73 (8.55–585.40)	205.09	95.1	< 0.001
Negative likelihood ratio (PLR)	0.43 (0.28–0.66)	99.77	90.0	< 0.001
Diagnostic odd ratio (DOR)	193.06 (51.21–727.83)	36.66	72.7	< 0.001
**EPTB** **(*n*** **= 10;** ^b^ **2327)** **AUC=0.99**		X^2^ (d.f. = 9)	l^2^	*p* value
Sensitivity	87(82–91)	47.45	81.00	< 0.001
Specificity	100(99–100)	19.19	53.10	0.0236
Positive likelihood ratio (PLR)	111.91(53.97–232.04)	11.74	23.40	0.2282
Negative likelihood ratio (PLR)	0.15 (0.07–0.30)	29.44	69.40	0.0005
Diagnostic odd ratio (DOR)	1337.84 (441.92–4050.12)	13.02	30.90	0.1610

^a^Random effects model, *χ*2 chi-squared, *d.f.* degree of freedom, *I*^2^ *I*-squared. ^b^ number of specimens, *n* number of studies, *CI* confidence interval, *AUC* area under receiver operating characteristics curve, *PTB* pulmonary tuberculosis, *EPTB* extra-pulmonary tuberculosis

### Subgroup analyses by impact of RT-PCR based assay on countries

Subgroup analyses by Impact of RT-PCR based assays on lower- and middle-income countries (LMICs) versus upper middle-income countries (UMICs). We assessed sources of data to these graders.

1. With LMICs (Table 1) as the RT-PCR based assay (8 studies, 2,047 specimens), the results were as follows: sensitivity 65 (95% CI 58–72), specificity 99 (95% CI, 99–99) and AUC 0.99. A test with perfect discrimination has a ROC curve that passes through the upper left corner (100% sensitivity, 100% specificity). The closer the ROC curve to the upper left corner, the higher the overall accuracy of the test. The summary estimates of performance of RT-PCR based assay in LMICs heterogeneity with chi-squared (*χ*2) using 95% CI for sensitivity, specificity, PLR, NLR and DOR were 44.28, 10.46, 5.97, 29.54 and 11.25, respectively, and *p =* < 0.001–0.5 indicating significant heterogeneity across studies. *I* ^2^ was between 37.8 and 84.20% showing significant heterogeneity. The results for subgroup analysis by RT-PCR based assay in LMICs are as presented in Table 3 and Additional file 4 and show considerable heterogeneity.

**Table 3:** Subgroup analyses by impact of RT-PCR based assays on lower- and middle-income countries (LMICs) versus upper middle-income countries (UMICs). We will assess sources of data to these graders

Test property	Summary of measure test accuracy^a^ (95%)	Test of heterogeneity		
**Lower-MICs** **(*n*** **= 8;** ^b^ **2047)** **AUC = 0.99**		*χ*2 (d.f. = 7)	*l*^*2*^	*p* value
Sensitivity	65 (58–72)	44.28	84.20	< 0.001
Specificity	99 (99–99	10.46	33.10	0.1639
Positive likelihood ratio (PLR)	86.61 (46.72–160.53)	5.97	0.0	0.5432
Negative likelihood ratio (NLR)	0.367 0.233–0.578	29.54	76.30	< 0.001
Diagnostic odd ratio (DOR)	311.43 (106.76–908.51)	11.25	37.8	0.1280
**Upper-MICs** **(*n*** **= 12;** ^b^ **3487)** **AUC = 0.99**		*χ*2 (d.f. = 11)	*l*^*2*^	*p* value
Sensitivity	68 (63–73)	197.71	94.40	< 0.001
Specificity	97 (96–98)	291.40	96.20	< 0.001
Positive likelihood ratio (PLR)	80.90 (10.31–634.9)	247.81	95.6	< 0.001
Negative likelihood ratio (NLR)	0.20 (0.09–0.42)	228.53	95.2	< 0.001
Diagnostic odd ratio (DOR)	522.72 (107.04–2552.8)	50.80	78.30	< 0.001

^a^Random effects model, *χ*2 chi-squared, *d.f.* degree of freedom, *I*^2^ *I*-squared, ^b^ number of specimens, *n* number of studies, *CI* confidence interval, *AUC* area under receiver operating characteristics curve, *PTB* pulmonary tuberculosis, *EPTB* extra-pulmonary tuberculosis

2. With UMICs (Table 1) as the RT-PCR based assay (12 studies, 3489 specimens), the results were as follows: sensitivity 68 (95% CI 63–73), specificity 97 (95% CI 96–98) and AUC 0.99. A test with perfect discrimination has a ROC curve that passes through the upper left corner (100% sensitivity, 100% specificity). The closer the ROC curve to the upper left corner, the higher the overall accuracy of the test. The summary estimates of performance of RT-PCR based assay in UMICs heterogeneity with chi-squared (*χ*2) using 95% CI for sensitivity, specificity, PLR, NLR and DOR were 197.71, 291.40, 247.81, 228.53 and 50.80, respectively, and *p =* < 0.001 indicating significant heterogeneity across studies. *I*^2^ was between 37.8 and 84.20% showing significant heterogeneity. The results for subgroup analysis by RT-PCR based assay in UMICs are as presented in Table 3 and Additional file 5 and show considerable heterogeneity.

## Discussion

Tuberculosis is a global health threat, and early and accurate diagnosis is crucial for preventing morbidity and mortality. Various methods are employed for the diagnosis of TB such as smear microscopy, culture identification, histopathology, tuberculin skin test (TST), serological assays, interferon-gamma release assays (IGRAs) and nucleic acid amplification (NAA) tests [64, 65]. The main advantages of RT-PCR based assay are shortened turn-a-round time, quantification of bacterial load and automation of the procedure that reduces hands-on time and decreased risk of cross-contamination [66, 67].

This review provides evidence on the paediatric tuberculosis diagnosis using *Mycobacterium tuberculosis* RT-PCR based assay for the rapid and accurate detection of MTB from clinical samples. To our knowledge, this is the first systematic review and meta-analysis for ascertaining the advantage of RT-PCR based assays for the detection of paediatrics MTB from both pulmonary and extra-pulmonary clinical samples. This systematic review and meta-analysis are broader in scope and included other types of RT-PCR based assay other than Xpert MTB/RIF than a previous meta-analysis on this topic [68] and the inclusion of data from low-, middle- and high-income countries.

In this study, results indicated that RT-PCR based assay produces consistent results with high specificity of 97 (95% CI 96–98), PLR of 70.73 (8.55–585.40) and NLR of 0.43 (0.28–0.66) for PTB, whereas specificity, PLR and NLR were 100 (95% CI 99–100), 111.91 (53.97–232.04) and 0.15 (0.07–0.30), respectively, for EPTB. A PLR of 71 suggests that patients with a pulmonary MTB infection have a 71-fold higher chance of being RT-PCR-based test positive compared with patients without the infection. This ratio suggests a potential role for RT-PCR assay in confirming (ruling in) an MTB infection in paediatrics.

The summary estimates of sensitivity, however, were 56 (95% CI 51–62) and 87 (95% CI 82–91) for pulmonary and extra-pulmonary samples, respectively, higher in extra-pulmonary than pulmonary TB possibly due to quality and paucity of tubercle bacilli in paediatrics sputum samples. Sensitivity estimates were more variable than specificity. According to the AUC and the DOR (see Table 2), diagnostic accuracy of RT-PCR based assay was excellent for the extra-pulmonary specimens over pulmonary and these results are acceptable for clinical practice (see Table 2).

A RT-PCR based assay for the detection of MTB has a high sensitivity and specificity. The PLR and NLR showed that RT-PCR may serve as a suitable method when confirming or excluding TB. It was anticipated that there would be some degree of heterogeneity of diagnostic measures across studies due to differences in sample size, RT-PCR based assay type, reference test of mycobacterial culture (either liquid or solid or both) and TB prevalence. High heterogeneity was found among studies (as defined by the *χ*2 and *I*^2^ statistics) for all measures. Subgroup analyses were therefore performed pre-specified to investigate potential sources of the observed between-study heterogeneity. It was assumed that the disparity was likely a result of the differences in the type of index test (RT-PCR assay) or target sequence gene of MTB used or the income categories of countries in the included studies.

In the current study, a limited number of subgroup analyses were conducted by comparing the impact of RT-PCR based assays on categories of lower- and middle-income countries (LMICs) versus upper middle-income countries (UMICs) to reduce the degree of study heterogeneity. Heterogeneity assessed by *χ*2 and *I*^2^ statistics between these subgroups was generally not very strong (see​Table 3). However, significant heterogeneity of diagnostic accuracy measures was expected and was, indeed, found among studies and the random-effects model partially accounted for the between-study heterogeneity.

Some degree of heterogeneity of diagnostic measures across studies was found due to differences in sample size, sample type, study design, target genes and clinical settings of the participants. Thus, it is possible that when evaluating RT-PCR assays using a more sensitive index test can lead to overestimation of the assay’s sensitivity.

## Strengths and weaknesses of the review

An important strength of this study was its comprehensive search strategy using several search engines to identify any unpublished studies in the form of conference abstracts or proceedings. Screening, study selection, quality assessment and data extraction were undertaken independently and reproducibly by two reviewers, as such human error should be limited. The problem of missing data was reduced by contacting the authors of the publications. In accordance with the study guidelines, potential publication bias and heterogeneity was explored [69, 70]. Random-effects analysis and subgroup analyses in anticipation of heterogeneity were used. Evaluation of level of publication bias was not formally carried out in the study; however, the risk of this bias was reduced by not restricting the search to any language. Another strength of this review is that RT-PCR based assay has comparably high sensitivity with paucibacillary specimens (EPTB) and high throughput capacities particularly in paediatrics where getting quality sputum samples is difficult.

This review does, however, have some limitations in assessing issues such as cost-effectiveness and the net effect of RT-PCR assay on clinical care and patient outcomes. Also, because of poor reporting, an analysis of the effect of factors such as laboratory infrastructure was not possible. Secondly, empirical evidence suggests that studies with significant or favourable results are more likely to be published than those with non-significant or unfavourable results [71]. In addition, literature search strategies are inherently imperfect, and studies can be missed, it is therefore possible that a proportion of such studies with non-significant or unfavourable results may have been missed. Other limitations are conflicts of interest of study authors particularly from industry supported studies and fully keeping up to date with the primary studies in this rapidly evolving field. Two of the included studies in our analysis were case-control, and thus subject to potential bias that could have affected outcomes.

Given that RT-PCR based assays in this review cover a range of different target genes and procedures, it is not possible to recommend any one over another owing to a lack of direct test comparisons. Our findings should be interpreted in the context of the quality of studies and reporting and variability in study quality. Diagnostic studies in general [72] and TB diagnostic studies in particular [73, 74] seem to be beset by these problems.

The use of guidelines such as the Standards for Reporting of Diagnostic Accuracy (STARD) might improve the quality of reporting of primary studies [75]. Future studies should compare commercialised RT-PCR assays to determine their diagnostic accuracy. Further work is required to devise a simple and cost-effective RT-PCR test for an efficient diagnosis of PaeTB that can be used routinely in resource-poor countries.

## Conclusion

According to this review and meta-analysis, RT-PCR assay has a high sensitivity and specificity for EPTB with turn-a-round time of 2 h compared with reference culture-based assay that takes between 2 and 10 weeks for detection. Supporting the fact that where quality pulmonary (sputum) samples could not be collected in paediatrics, the use of extra-pulmonary samples should be considered. Overall, RT-PCR based assay accuracy was superior for extra-pulmonary samples (sensitivity 87 (95% CI 82–91); specificity 100 (95% CI 99–100) as opposed to pulmonary samples sensitivity 56 (95% CI 51–62); specificity 97 (95% CI 96–98). The specificity was high for both pulmonary and extra-pulmonary samples indicating that the test should be adopted as the first-line test for ruling in TB infection but may need to be an add-on test to rule out the disease. It offers an alternative robust approach to detect MTB in paucibacillary PaeTB samples, showing rapid results with good diagnostic accuracy. The results of our study should provide encouragement to health-care providers for treating children with TB. Nevertheless, the results of this assay should be interpreted in parallel with clinical findings and the results of conventional tests, but the assay may contribute significantly for an early diagnosis and exert an impact on the clinical management and control of TB. The findings do not support the use of this assay for excluding a diagnosis of TB on its own as a standalone test. It offers an incremental benefit as an add-on test to other investigations. RT-PCR assays, combining amplification and detection in a single run, seem to offer advantages over conventional assays including the mycobacterial culture-based reference standard which is slow.

It is anticipated that our findings will aid healthcare practitioners and policymakers in adopting the use of this assay on a routine basis. Most importantly, this can be as a point-of-care-test which will help in the global control of PaeTB, particularly in developing countries with a high burden of the disease.

## Supplementary Information

**Additional file 1.** PRISMA checklist.

**Additional file 2.** Search strategy.

**Additional file 3.** Quality assessment of diagnostic accuracy studies-2 tool.

**Additional file 4.** Figures of Subgroup analyses (LMICs).

**Additional file 5.** Figures of Sub-group analyses (UMICs).

**Additional file 6.** Definition of statistical parameters.

## Data Availability

The datasets used and/or analysed during the current study are available from the corresponding author on reasonable request.
