# Supplementary material for: Paediatric tuberculosis diagnosis using Mycobacterium tuberculosis real-time polymerase chain reaction assay: a systematic review and meta-analysis
Source: Syst Rev. 2021 Oct 27;10:278. doi: 10.1186/s13643-021-01836-w (PMC8554997; doi:10.1186/s13643-021-01836-w)
Supplement: Supplementary file 2 — Additional file 2. Search strategy. [file 13643_2021_1836_MOESM2_ESM.docx]

**Additional file 2:**

**ADDITIONAL FILE 2: Search strategy**

The following Medline via PubMed search algorithm will be translated to EMBASE**:**

1. ("tuberculosis") ti,ab

2. (mycobacterium tuberculosis) ti,ab

3. (extrapulmonary tuberculosis) ti,ab

4. (pulmonary tuberculosis) ti,ab

5. (paediatric tuberculosis) ti,ab

6. 1 OR 2 OR 3 OR 4 OR 5

7. ("Real-time polymerase chain reaction") ti,ab

8. (real-time pcr) ti,ab

9. (real-time pcr assay) ti,ab

10. ("rt-pcr") ti,ab

11. ("Nucleic Acid Amplification Test") ti,ab

12. ("NAAT") ti,ab

13. 7 OR 8 OR 9 OR 10 OR 11 OR 12

14. ("culture-based media") ti,ab

15. (culture-based assay) ti,ab

16. ("liquid media") ti,ab

17. ("solid media") ti,ab

18. 14 OR 15 OR 16 OR 17

19. (“paediatric”) ti,ab

20. (“paediatrics”) ti,ab

21. (“children”) ti,ab

22. 19 OR 20 OR 21

23. 6 AND 13

24. 18 AND 22

25. 23 AND 24
