# Supplementary material for: Paediatric tuberculosis diagnosis using Mycobacterium tuberculosis real-time polymerase chain reaction assay: a systematic review and meta-analysis
Source: Syst Rev. 2021 Oct 27;10:278. doi: 10.1186/s13643-021-01836-w (PMC8554997; doi:10.1186/s13643-021-01836-w)
Supplement: Supplementary file 4 — Additional file 4. Figures of Subgroup analyses (LMICs). [file 13643_2021_1836_MOESM4_ESM.docx]

**Additional file 4** Figures of Subgroup analyses (LMICs)

**Figure S1** Forest plot estimates of the pooled sensitivity for LMICs

 **Figure S2** Forest plot estimates of the pooled specificity for LMICs

**Figure S3** Forest plot estimates of the pooled PLR for LMICs

**Figure S4** Forest plot estimates of the pooled NLR for LMICs

**Figure S5** Forest plot estimates of the pooled DOR for LMICs

**Figure S6** Forest plot estimates of the pooled SROC for LMICs
