# Supplementary material for: Paediatric tuberculosis diagnosis using Mycobacterium tuberculosis real-time polymerase chain reaction assay: a systematic review and meta-analysis
Source: Syst Rev. 2021 Oct 27;10:278. doi: 10.1186/s13643-021-01836-w (PMC8554997; doi:10.1186/s13643-021-01836-w)
Supplement: Supplementary file 5 — Additional file 5. Figures of Sub-group analyses (UMICs). [file 13643_2021_1836_MOESM5_ESM.docx]

**Additional file 5: Figures of Sub-group analyses (UMICs)**

**Figure S1** Forest plot estimates of the pooled sensitivity for UMICs

 **Figure S2** Forest plot estimates of the pooled specificity for UMICs

 **Figure S3** Forest plot estimates of the pooled PLR for UMICs

 **Figure S4** Forest plot estimates of the pooled NLR for UMICs

 **Figure S5** Forest plot estimates of the pooled DOR for UMICs

 **Figure S6** Forest plot estimates of the pooled SROC for UMICs
